# DeepMHCII: a novel binding core-aware deep interaction model for accurate MHC-II peptide binding affinity prediction

**DOI:** 10.1093/bioinformatics/btac225

**Published:** 2022-06-27

**Authors:** Ronghui You, Wei Qu, Hiroshi Mamitsuka, Shanfeng Zhu

**Affiliations:** Institute of Science and Technology for Brain-Inspired Intelligence and MOE Frontiers Center for Brain Sciences, Fudan University, Shanghai 200433, China; Institute of Science and Technology for Brain-Inspired Intelligence and MOE Frontiers Center for Brain Sciences, Fudan University, Shanghai 200433, China; Shanghai Qi Zhi Institute, Shanghai 200030, China; Bioinformatics Center, Institute for Chemical Research, Kyoto University, Uji, Kyoto Prefecture, Japan; Department of Computer Science, Aalto University, Espoo, Finland; Institute of Science and Technology for Brain-Inspired Intelligence and MOE Frontiers Center for Brain Sciences, Fudan University, Shanghai 200433, China; Shanghai Qi Zhi Institute, Shanghai 200030, China; Key Laboratory of Computational Neuroscience and Brain-Inspired Intelligence (Fudan University), Ministry of Education, Shanghai 200433, China; Shanghai Key Lab of Intelligent Information Processing and Shanghai Institute of Artificial Intelligence Algorithm, Fudan University, Shanghai 200433, China; Zhangjiang Fudan International Innovation Center, Shanghai 200433, China; National Genomics Data Center, CAS Key Laboratory of Computational Biology, Bio-Med Big Data Center, Shanghai Institute of Nutrition and Health, University of Chinese Academy of Sciences, Chinese Academy of Sciences, Shanghai 200031, China

## Abstract

**Motivation:**

Computationally predicting major histocompatibility complex (MHC)-peptide binding affinity is an important problem in immunological bioinformatics. Recent cutting-edge deep learning-based methods for this problem are unable to achieve satisfactory performance for MHC class II molecules. This is because such methods generate the input by simply concatenating the two given sequences: (the estimated binding core of) a peptide and (the pseudo sequence of) an MHC class II molecule, ignoring biological knowledge behind the interactions of the two molecules. We thus propose a binding core-aware deep learning-based model, DeepMHCII, with a binding interaction convolution layer, which allows to integrate all potential binding cores (in a given peptide) with the MHC pseudo (binding) sequence, through modeling the interaction with multiple convolutional kernels.

**Results:**

Extensive empirical experiments with four large-scale datasets demonstrate that DeepMHCII significantly outperformed four state-of-the-art methods under numerous settings, such as 5-fold cross-validation, leave one molecule out, validation with independent testing sets and binding core prediction. All these results and visualization of the predicted binding cores indicate the effectiveness of our model, DeepMHCII, and the importance of properly modeling biological facts in deep learning for high predictive performance and efficient knowledge discovery.

**Availability and implementation:**

DeepMHCII is publicly available at https://github.com/yourh/DeepMHCII.

**Supplementary information:**

Supplementary data are available at *Bioinformatics* online.

## 1 Introduction

Major histocompatibility complex (MHC) molecules play a significant role in the T-cell mediated adaptive immune response (Janeway *et al.*, 2005). MHC molecules first bind peptide fragments derived from pathogens and then present the peptides to the surface of antigen-presenting cells (APC). After the MHC-peptide complexes are recognized by T-cell receptors (TCR), adaptive immune response will be stimulated to fight against and eliminate invading pathogens. Accurate identification of MHC binding peptides is thus crucial for not only elucidating the mechanism of immune recognition but also facilitating the design of peptide-based vaccine and cancer immunotherapy (Blass and Ott, 2021). As biochemical experiments are time consuming and labor intensive, computational approaches for predicting MHC binding peptides have become increasingly important and have been utilized to prioritize a small number of promising candidates for further verification by biochemical experiments (Hu *et al.*, 2010; Lund *et al.*, 2005; Mamitsuka, 1998; Udaka *et al.*, 2002; Zhu *et al.*, 2006).

There are two major classes of MHC molecules: MHC Class I (MHC-I) and MHC Class II (MHC-II). MHC-I molecules have one chain (*α*) and MHC-II molecules have two chains (*α* and *β*). Human MHC-II molecules are encoded in the human leucocyte antigen (HLA) gene complex involving three types of molecules: DP, DQ, DR, while mouse MHC-II are encoded in the histocompatibility 2 (H-2) (Traherne, 2008). MHC-I and MHC-II molecules play different roles in adaptive immune response. MHC-I molecules bind a rather fixed length of short peptides (usually 8–11 amino acids) from endogenous antigen, and these peptides are presented to cytotoxic T lymphocytes. In contrast, MHC-II molecules bind a more diverse length of peptides from exogenous antigen, and these peptides are presented to helper T lymphocytes. It has also been reported that MHC-II peptide binding involves the B-cell mediated adaptive immune response. From these aspects, predicting peptides binding to MHC-II is more challenging than that of MHC-I (Nielsen *et al.*, 2010). Currently, the state-of-the-art methods for MHC-I peptide binding prediction can achieve an area under the ROC curve (AUC) of around 0.9, while AUC by the prediction methods for MHC-II is usually unable to reach 0.8, particularly for MHC-II molecules with very few or no binding peptide data (Jensen *et al.*, 2018; Zhang *et al.*, 2012b), which is far from satisfactory. Moreover, quantitative prediction of MHC peptide binding is more useful in practice than binary classification for selecting a small number of promising candidate peptides. It is thus imperative to develop accurate computational methods for MHC-II peptide binding affinity prediction.

The challenges come from two sides: peptides and MHC-II molecules. For the peptide side, the binding groove of MHC-II molecules is open at both ends, which causes large variation on the length of binding peptides, ranging from 10 to 30 amino acids (typically 12–16). The binding groove of MHC-II has nine pockets, where one amino acid residue of the binding core of a binding peptide fits to one pocket normally (Janeway *et al.*, 2005). The peptide-MHC binding affinity is primarily determined by the interaction between MHC-II molecules and the binding core of peptides. However, it has been found that peptides flanking regions (PFRs) which are outside of the binding core also affect the binding affinity (Arnold *et al.*, 2002; Holland *et al.*, 2013). Thus, there are two issues for the peptide side: (i) the flexibility in length and (ii) the location of the binding core. For the MHC-II molecule side, MHC-II molecules are highly polymorphic. There are thousands of MHC-II molecules, and each MHC-II molecule has its own binding specificity. Also, MHC molecules are proteins to be represented by amino acid sequences with longer and more diverse lengths than peptides. In addition, currently, only dozens of MHC-II molecules have hundreds of binding affinity data in the immune epitope database and analysis resource (IEDB) (Vita *et al.*, 2019), and a vast majority of MHC-II molecules have very few or even no binding data. Thus, there are three issues for the MHC-II side: (i) thousands of MHC-II molecules with different binding specificity, (ii) long and size-flexible sequences and (iii) data scarcity for most of the MHC-II molecules. These aspects have made it difficult to model the interaction of MHC-II peptide binding accurately.

Computational methods for MHC-II peptide binding prediction can be divided into two categories: allele-specific and pan-specific (Zhang *et al.*, 2012b). Allele-specific methods can predict only binding preferences of MHC-II molecules in the training set, while pan-specific methods can predict binding preferences of MHC-II molecules even with no training data of these molecules, which is thus the focus of our work. Traditionally, pan-specific methods have been developed by various different techniques, such as position-specific scoring matrices (Zhang *et al.*, 2012a), artificial neural network (ANN) (Jensen *et al.*, 2018), kernel-based methods (Guo *et al.*, 2013) and ensemble learning (Xu *et al.*, 2016). The most established method is NetMHCIIpan [latest version for MHC-II peptide binding prediction is NetMHCIIpan-3.2 (Jensen *et al.*, 2018)], an ANN-based method, which pioneered using pseudo sequence to represent an MHC-II molecule. ANN deals with only a fixed-sized input, and so NetMHCIIpan first estimates the binding core in a given peptide and then trains an ANN using the estimated binding cores and the pseudo sequences. The whole process is repeated until convergence. However, the first estimation of the binding core might be inaccurate, which affects the predictive performance heavily. Most recent pan-specific methods use deep learning (DL), such as convolutional neural network (CNN), long short-term memory and transformer to learn the interaction between MHC-II molecules and peptides. There exist four representative DL-based methods: PUFFIN (Zeng and Gifford, 2019), DeepSeqPanII (Liu *et al.*, 2021), MHCAttnNet (Venkatesh *et al.*, 2020) and BERTMHC (Cheng *et al.*, 2021). In spite of using advanced DL techniques, these methods concatenate the sequence encoding of an MHC-II molecule and a peptide for the input, which do not take advantage of important domain knowledge, resulting in the lacking of performance improvement and interpretability.

We propose a novel deep learning-based method, DeepMHCII, for accurate MHC-II peptide binding affinity prediction by incorporating biological knowledge into designing the model architecture. Specifically, DeepMHCII is modeled, considering the following three distinct features: (i) binding core and PFRs in each peptide; (ii) pseudo sequence, i.e. the sequence with only crucial residues for directly interacting with the binding core of the counterpart peptide, in each MHC-II molecule. (iii) Interaction between a peptide and an MHC-II molecule by the interaction between all potential binding cores and the pseudo sequence. Note that these three features have not been explicitly addressed by any existing methods simultaneously. Specifically, DeepMHCII generates a binding interaction convolutional layer (BICL) with adaptive kernel size filters to model the interaction between peptides and MHC-II molecules.

The performance of DeepMHCII has been thoroughly validated by extensive experiments on four benchmark datasets under various settings, such as 5-fold cross-validation, leave one molecule out (LOMO), independent testing set verification and binding core prediction. We compared the predictive performance of DeepMHCII with four state-of-the-art methods: NetMHCIIpan-3.2 (Jensen *et al.*, 2018), PUFFIN (Zeng and Gifford, 2019), DeepSeqPanII (Liu *et al.*, 2021) and MHCAttnNet (Venkatesh *et al.*, 2020). Experimental results demonstrate that DeepMHCII outperformed all competing methods in all experiments. The improvement was especially significant in LOMO and independent testing set verification. For example, DeepMHCII achieved an average AUC of 0.77 in independent testing, which was 7% and 10% higher than that of NetMHCIIpan-3.2 (0.719) and PUFFIN (0.70). In addition, DeepMHCII achieved an average Pearson correlation coefficient (PCC) of 0.560 in LOMO, which was 6.7% higher than that of PUFFIN (0.525). All these results indicate the effectiveness of DeepMHCII on predicting the binding specificity of unseen MHC-II molecules. We also verified the performance advantage and interpretability of DeepMHCII in identifying the binding core of peptides and binding motifs of MHC-II molecules.

## 2 Methods

### 2.1 Problem formulation

Suppose $P=(p_{1},p_{2},\ldots ,p_{L})$ denotes the peptide sequence and $Q=(q_{1},q_{2},\ldots ,q_{L\prime })$ denotes the L′-length MHC-II molecule sequence, where each of *p_i_* and *q_j_* stands for one of the 20 types of amino acids. The task is a regression problem to predict the binding affinity $\hat{z}\in [0,1]$ by a given pair of *P* and *Q*. The binding affinity is mainly determined by the nine-length binding core (unknown) of the peptide and the binding groove with nine pockets in the MHC-II molecule. In addition, it has been found that peptides flanking regions (PFRs) of the binding core affect the binding affinity.

In practice, we use the 34-length pseudo sequence Q′ extracted from *Q* as the representation of MHC-II molecules. Pseudo sequence of MHC-II refers to the important MHC residues that are considered being crucial for peptide binding. The 34-length MHC-II molecule pseudo sequence was first proposed in NetMHCIIpan-3.0 (Karosiene *et al.*, 2013), which was composed of 15 and 19 amino acid residues from *α* and *β* chains of MHC-II, respectively. These residues were extracted from MHC-II peptide complexes in PDB (Burley *et al.*, 2021), which were polymorphic in MHC molecules and found in close contact (<4 Å) with peptide binding core in at least one MHC-II peptide complex. The 34-length MHC-II pseudo sequence has also been used in NetMHCIIpan-3.1 (Andreatta *et al.*, 2015) and 3.2 (Jensen *et al.*, 2018). Similarly, we use this pseudo sequence as the representation of MHC-II molecules.

### 2.2 Overview

**Fig. 1.**
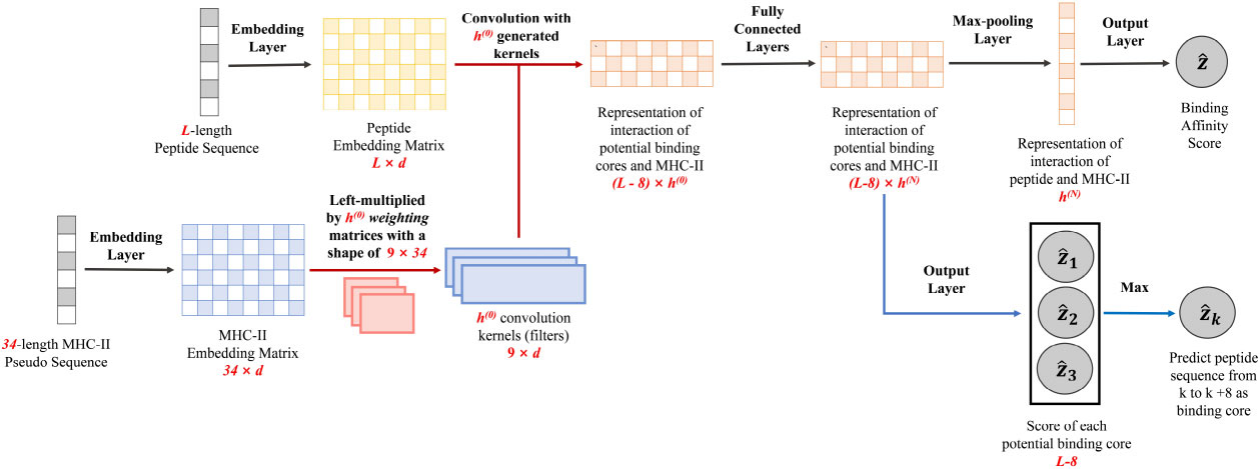
The architecture of DeepMHCII. The red arrows are processes of the binding interaction convolutional layer (BICL) and the blue arrows are processes of binding core prediction.

The basic idea of DeepMHCII is (i) to use deep learning to explicitly model the interaction between an MHC-II molecule sequence and a peptide and (ii) considering the crucial residue for binding, to focus on only the interaction between the binding core in a peptide and counterpart important amino acid residues (pseudo sequence) in a MHC-II molecule. Specifically, the input of DeepMHCII is a *L*-length peptide sequence *P* and, a 34-length pseudo sequence Q′ extracted from an MHC-II molecule sequence *Q* (Burley *et al.*, 2021; Jensen *et al.*, 2018). Figure 1 shows the architecture of DeepMHCII with mainly four steps: (i) we apply an embedding layer to the peptide sequence and also another embedding layer to the pseudo sequence of MHC-II to obtain deep semantic dense representations; (ii) we use a binding interaction convolutional layer (BICL) to obtain the representation of binding interaction between the potential binding cores and the MHC-II molecule; (iii) we use fully connected layers and a max-pooling layer to extract the interaction of peptide and MHC-II molecule; and (iv) we use an output layer to obtain the predicted binding affinity.

### 2.3 Input layer

We use an embedding layer to encode amino acid sequences for peptides and also similarly another embedding layer for MHC-II pseudo sequences. Let *L* be the length of an input peptide and *d* be the dimension of amino acid embeddings.

Then, for a given pair of a peptide sequence *P* and an MHC-II molecule pseudo sequence $Q\prime ,\;X\in R^{L\times d}$, the output of the embedding layer for *P*, and $Y\in R^{34\times d}$, the output of the embedding layer for Q′, are given as follows:

$$X={\left(x_{1},x_{2},\ldots ,x_{L}\right)}^{T},\;Y={\left(y_{1},y_{2},\ldots ,y_{34}\right)}^{T},$$

where $x_{i}\in R^{d}$ and $y_{j}\in R^{d}$ are the representation of the *i*th amino acid of the peptide sequence and the *j*th amino acid of the MHC-II pseudo sequence, respectively.

### 2.4 Binding interaction convolutional layer

We use a binding interaction convolutional layer (BICL) to obtain the representation of the interaction between peptide **X** and MHC-II molecule **Y** by considering all possible binding cores of **X**. Traditionally in sequence-based CNN, input sequences share the same kernels (filters), while in our problem, each MHC-II molecule has a distinguished binding preference. To address this issue, BICL generates different kernels for each MHC-II molecule. Specifically, letting *M* be the size of a binding core (=9 in our problem), we use a weight matrix $W_{k}$ with the size of M×34 to generate the *k*th kernel as $f(W_{k}Y)$, where *f* is the activation function. We can write a potential binding core of **X** starting with the *i*th residue as $X_{i:i+M-1}$. By using $X_{i:i+M-1}$ and kernel $f(W_{k}Y)$, the interaction between potential binding core $X_{i:i+M-1}$ and the MHC-II molecule **Y** can be given as follows:

$$C_{i,k}^{\left(0\right)}=f(f(W_{k}Y)\cdot X_{i:i+M-1}+b_{k}),$$

where $b_{k}$ is the bias. Then the output of BICL can be written as $C^{\left(0\right)}\in R^{(L-M+1)\times h^{\left(0\right)}}$, where $h^{\left(0\right)}$ is the number of kernels.

Considering the effect of both binding core and PFRs, we used four different kernel sizes: 9, 11, 13 and 15. For example, kernel size of 15 is used to consider the effect of three more amino acids in the left and right side of binding core, respectively. For each kernel size, we used a different number of kernels. That is, the number of kernels for the kernel size of nine was the largest, followed by those of 11, 13 and 15. This setting will be described in detail in Section 3.2. Note that the padding symbol will be added to both side of **X** for kernel sizes other than 9 to get the same number of rows (number of potential binding cores) of output.

### 2.5 Max-pooling and output layer

We then use *N* fully connected layers and a max-pooling layer to obtain the representation $g\in R^{h^{\left(N\right)}}$ of the interaction between peptide **X** and MHC-II molecule **Y** as follows:

$$\begin{array}{c} C_{i}^{\left(n\right)}=f(C_{i}^{\left(n-1\right)}W^{\left(n\right)}+b^{\left(n\right)}),\\ g_{j}=\max\{C_{1,j}^{\left(N\right)},C_{2,j}^{\left(N\right)},\ldots ,C_{L-M+1,j}^{\left(N\right)}\}, \end{array}$$

where 1≤n≤N, and $W^{\left(n\right)}\in R^{h^{\left(n-1\right)}\times h^{\left(n\right)}},\;b^{\left(n\right)}\in R^{h^{\left(n\right)}}$ and $C^{\left(n\right)}\in R^{(L-M+1)\times h^{\left(n\right)}}$ are the weights, bias and output of the *n*th fully connected layer, respectively.

Finally, we use the output layer to obtain the predicted binding affinity $\hat{z}\in [0,1]$ as follows:

$$\hat{z}=\sigma (w^{\left(o\right)}\cdot g+b^{\left(o\right)}),$$

where $w^{\left(o\right)}\in R^{h^{\left(N\right)}}$ and $b^{\left(o\right)}\in R$ are the weights and bias, respectively. Our training objective is to minimize the mean square error. Practically, we trained *T* models with different random initial weights and used the average over the *T* predicted scores as the final prediction.

### 2.6 Binding core prediction

For a given pair of peptide sequence and MHC-II molecule, we remove max-pooling layer of DeepMHCII, use the output layer after *N* fully connected layers directly to obtain the predicted score of each nine-length potential binding core as follows:

$${\hat{z}}_{i}=\sigma (w^{\left(o\right)}\cdot C_{i}^{\left(N\right)}+b^{\left(o\right)}),$$

The nine-length subsequence of peptide with the highest score (${\hat{z}}_{k}$) will be recommended as the binding core of this pair.

## 3 Experiments

### 3.1 Datasets

We used four publicly available benchmark datasets (BD2016, ID2017, BD2020 and BC2015) to train and evaluate DeepMHCII and competing methods: BD2016 and ID2017 for MHC-peptide binding affinities, BD2020 for MHC-peptide binding classification and BC2015 for predicting binding cores. Below, we describe more details on each of the four datasets.

BD2016 (http://www.cbs.dtu.dk/suppl/immunology/NetMHCIIpan-3.2): BD2016 contains 134 281 data points of MHC-peptide binding affinities over 80 different MHC-II molecules, including 36 HLA-DR, 27 HLA-DQ, 9 HLA-DP and 8 H-2 molecules. BD2016 was compiled for training NetMHCIIpan-3.2 (Jensen *et al.*, 2018) from IEDB. The original, experimentally obtained IC50 binding value of each data point was transformed into the binding affinity with the range of [0,1] by $1-\text{log}({\text{IC}}_{50}nM)/\;\text{log}(50,000)$. BD2016 already provides a 5-fold cross-validation (5-fold CV) split which groups the peptides with common motifs into the same fold. Table 1 shows a summary of BD2016.

**Table 1.** Summary statistics of BD2016

Allele	No. of peptides	No. of binders	No. of MHCs
HLA-DR	87 363	40 756	36
HLA-DP	15 564	5135	9
HLA-DQ	28 081	9098	27
H-2	3273	894	8
Total	134 281	55 883	80

ID2017: a dataset of MHC-peptide binding affinities was compiled from IEDB in 2017 for evaluating different MHC-II binding peptide prediction methods (Andreatta *et al.*, 2018). From this dataset, we generated an independent test dataset, ID2017, by removing data points overlapped with BD2016 and retaining MHC-II molecules with more than 50 peptides for robust performance evaluation. There are 10 HLA-DB molecules with 857 peptides in ID2017.

BD2020: a binary classification dataset of MHC-II peptide binding, which was extracted by Venkatesh *et al.* (2020) from IEDB for training MHCAttnNet. Note that BD2020 has no quantitative binding affinities. BD2020 consists of 65 954 data points for 49 HLA-DRB molecules, where 36 035 are positive, 28 919 are negative and 5-fold CV split is also provided.

BC2015: a binding core benchmark, which was used to evaluate the performance of NetMHCIIpan-3.2 in identifying the binding core of an MHC-peptide complex. BC2015 consists of 51 complexes from PDB.

We have calculated the following two probabilities to evaluate the redundancy of the 5-fold CV split of two benchmark datasets, BD2016 (redundancy-reduced partition) and BD2020 (random partition). Outer-p is the probability that two peptides in different folds have a common 9-mer subsequence (we focused on 9-mer, due to the binding core length), while Inner-p is the probability that two peptides in the same fold have a common 9-mer subsequence. As shown in Table 2, we can clearly see that BD2016 is less redundant, since Outer-p is only an around two percent of Inner-P for BD2016 while Outer-p and Inner-p have the same value for BD2020.

**Table 2.** Data redundancy of BD2016 and BD2020

Dataset	Outer-p	Inner-p	Ratio
BD2016	4.39e−4	2.41e−2	54.9
BD2020	2.43e−3	2.43e−3	1

### 3.2 Experimental settings

DeepMHCII used the following hyperparameter values, which were selected by 5-fold CV over BD2016: *d* (dimension of embeddings of amino acids) = 16. The numbers of kernels of BICL with the kernel sizes of 9, 11, 13 and 15 were 256, 128, 64 and 64, respectively. *N* (number of fully connected layers) = 2 and the sizes of nodes at the two layers were 256 and 128. *f* (activation function) was ReLU. We used batch normalization (Ioffe and Szegedy, 2015) after BICL and each of the fully connected layers. Also, we used dropout (Srivastava *et al.*, 2014) with the drop rate of 0.25 to avoid overfitting. During the training process, the batch size was 128, the number of epochs was 20 and the optimizer we used was Adadelta (Zeiler, 2012) with the learning rate of 0.9 and weight decay of 1e−4. *T* (number of trained models) was 20. We implemented DeepMHCII by PyTorch (Paszke *et al.*, 2019).

We compared DeepMHCII with four state-of-the-art methods: NetMHCIIpan-3.2 (http://www.cbs.dtu.dk/services/NetMHCIIpan-3.2) (Jensen *et al.*, 2018), PUFFIN (https://github.com/gifford-lab/PUFFIN) (Zeng and Gifford, 2019), DeepSeqPanII (https://github.com/pcpLiu/DeepSeqPanII) (Liu *et al.*, 2021) and MHCAttnNet (https://github.com/gopuvenkat/MHCAttnNet) (Venkatesh *et al.*, 2020). All are neural network-based methods. Since the training code of NetMHCIIpan-3.2 is unavailable, we used the experimental results (on BD2016) and the trained models provided by the authors directly. We trained PUFFIN and DeepSeqPanII on BD2016 using the implementation by the authors. According to the original paper and for a fair comparison, NetMHCIIpan-3.2 and both PUFFIN and DeepSeqPanII used a bagging ensemble of 200 and 20 models, respectively.

MHCAttnNet used the cross-entropy objective function and binary MHC-peptide binding dataset (BD2020) for model training. We then trained DeepMHCII with the same dataset and the cross-entropy objective function and compared the performance of MHCAttnNet obtained from the paper directly. Both DeepMHCII and MHCAttnNet used only one single model without any ensemble.

BERTMHC (Cheng *et al.*, 2021) also can be a competing method, while the open-source implementation (https://github.com/s6juncheng/BERTMHC) of BERTMHC was not consistent with the description in Cheng *et al.* (2021). We have then contacted the authors of BERTMHC, regarding this matter, but we were unable to receive any response (https://github.com/s6juncheng/BERTMHC/issues/8). As a result, we did not use BERTMHC in the performance comparison.

### 3.3 Evaluation metrics

We set up binary classification: we used the area under the receiver operating characteristics curve (AUC) for each MHC-II molecule and reported the average AUC. Also to classify peptides into binders and non-binders, a binding threshold of 500 nM was used: All peptides with an IC_50_ binding value < 500 nM (0.426 after transformation) were classified as binders. Since there are a large number of MHC molecules, we used a binomial test to check the statistical significance of performance difference (significance level was *P*-value < 0.05). In addition, we used the Pearson correlation coefficient (PCC) to examine the linear relationship between the predicted binding affinity and the true value.

### 3.4 Experimental results

We conducted the following four experiments to validate the predictive performance of DeepMHCII: (i) we examined the performance of DeepMHCII and the competing methods by 5-fold CV over BD2016. (ii) In order to validate the performance for unseen MHC-II molecules, following NetMHCIIpan-3.2, we conducted LOMO over BD2016 by using the above 5-fold CV set-up. Specifically, each time, data points of only one MHC-II molecule in the test fold were used for testing, while data points of all other MHC-II molecules in training folds were used for training over 5-fold CV settings. Furthermore, following the settings in NetMHCIIpan-3.2, out of all 81 MHC-II molecules, we focused on 61 molecules with more than 40 data points and at least three binders for the robustness of performance evaluation. (iii) We performed a performance comparison on DeepMHCII and competing methods on ID2017, i.e. the independent test set. (iv) We examined the performance of DeepMHCII using the same objective function as that of MHCAttnNet, to examine the robustness of DeepMHCII.

#### 3.4.1 Comparison of DeepMHCII and competing methods under 5-fold cross-validation

Table 3 reports the average AUC and PCC over all MHC-II molecules of 5-fold CV over BD2016 by DeepMHCII, NetMHCIIpan-3.2, PUFFIN and DeepSeqPanII (the detailed results of each MHC-II molecule are shown in Supplementary Table S1). DeepMHCII outperformed all competing methods in both AUC and PCC. For example, DeepMHCII achieved the highest AUC of 0.856, which was followed by NetMHCIIpan-3.2 (0.847), PUFFIN (0.846) and DeepSeqPanII (0.759). Also DeepMHCII outperformed NetMHCIIpan-3.2, PUFFIN and DeepSeqPanII on 48, 51 and 61, respectively, out of all 61 MHC-II molecules, all being statistically significant (two-tailed binomial test, *P*-value $=7.67\times 10^{-6},9.62\times 10^{-8},8.67\times 10^{-19}$, respectively).

**Table 3.** Performance of DeepMHCII and competing methods

Method	5-CV		LOMO		IEBD test	Binding core
	AUC	PCC	AUC	PCC	AUC	No. of correct/No. of total
NetMHCIIpan-3.2	0.847	0.679	0.775	0.544	0.719	45/51
PUFFIN	0.846	0.676	0.768	0.525	0.700	–
DeepSeqPanII	0.759	0.524	0.732	0.473	0.700	10/51
DeepMHCII	**0.856**	**0.691**	**0.785**	**0.560**	**0.770**	**47/51**

#### 3.4.2 Comparison of DeepMHCII and competing methods under LOMO

**Fig. 2.**
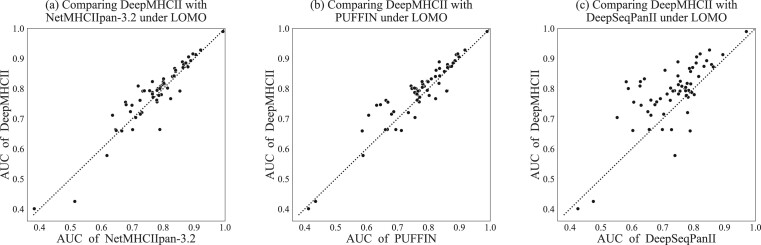
Performance comparison between DeepMHCII and (**a**) NetMHCIIpan-3.2, (**b**) PUFFIN and (**c**) DeepSeqPanII under LOMO. Each dot represents an MHC-II molecule

Also Table 3 reports the average AUC and PCC of LOMO over all MHC-II molecules by DeepMHCII and competing methods (the detailed results of each MHC-II molecule are shown in Supplementary Table S2). The results are consistent with those of 5-fold CV. DeepMHCII achieved the highest AUC and PCC of 0.785 and 0.560, respectively, which were 1.3% and 2.9%, respectively, higher than those achieved by NetMHCIIpan-3.2, the second-best method. Figure 2 plots the LOMO AUC results obtained by: DeepMHCII for y-axis and (Fig. 2a) NetMHCIIpan-3.2, (Fig. 2b) PUFFIN and (Fig. 2c) DeepSeqPanII for x-axis, where each dot corresponds to one MHC-II molecule. That is, if one dot appears above the diagonal line, DeepMHCII outperformed the competing method, regarding the MHC-II molecule corresponding to this dot. DeepMHCII outperformed NetMCHIIpan-3.2, PUFFIN and DeepSeqPanII on 43, 42, 51 out of all 61 molecules, respectively, all being statistically significant (two-tailed binomial test, *P*-value $=1.87\times 10^{-3},4.44\times 10^{-3}$ and $9.62\times 10^{-8}$, respectively). We also found that all methods tend to perform poorly on MHC molecules of H-2. In particular, there are several molecules such as H-2-IAs and H-2-IEk where DeepMHCII performs poorly. These may be related to the lack of similar MHC molecules, the relatively small amount of test data and the unknown quality of data. On the whole, these results indicate that DeepMHCII is more robust and can deal with unknown MHC-II alleles better than the competing methods.

Furthermore, we have reported the performance of DeepMHCII and PUFFIN over BD2016 under a more strict LOMO, where for each test MHC molecule, we removed the most similar molecules from the training sets to avoid ‘easy’ predictions. Detailed results are presented in the Supplementary material. We can see that the performance of DeepMHCII is still much better than PUFFIN. We did not use NetMHCIIpan-3.2 and DeepSeqPanII in this experiment, since no source codes of NetMHCIIpan-3.2 are provided and the performance of DeepSeqPanII is slower and worse than the other three methods.

#### 3.4.3 Comparison of DeepMHCII and competing methods on independent testing set

**Fig. 3.**
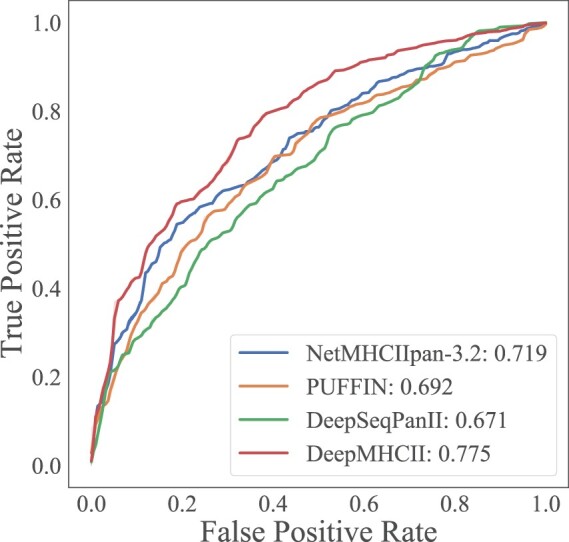
ROC curves by ID2017

Table 4 reports the performance on each MHC-II molecule of ID2017, the independent testing set, by DeepMHCII and competing methods. Figure 3 shows the ROC curves of DeepMHCII and competing methods on the whole ID2017. DeepMHCII outperformed all competing methods on both AUC of the whole ID2017 in Figure 3 and average AUC over all MHC-II molecules in Table 4. Specifically, DeepMHCII achieved the best average AUC of 0.770, which was 7.1% higher than the second-best method, NetMHCIIpan-3.2 (0.719), and more than 10% higher than the other two competing methods, PUFFIN and DeepSeqPanII. Regarding the AUC of the whole testing set, DeepMHCII achieved the highest AUC of 0.775, which was 7.8%, 12.0% and 15.5% higher than NetMHCIIpan-3.2 (0.719), PUFFIN (0.692) and DeepSeqPanII (0.671), respectively. For the performance over each MHC-II molecule, DeepMHCII outperformed NetMCHIIpan-3.2 and PUFFIN in all 10 MHC-II molecules and DeepSeqPanII in 8 out of the ten MHC-II molecules.

**Table 4.** Performance (AUC) of DeepMHCII and competing methods on the independent testing set

Allele	No. of peptides	No. of binders	NetMHCIIpan-3.2	PUFFIN	DeepSeqPanII	DeepMHCII
DBR1*01:01	100	81	0.880	0.834	0.733	**0.882**
DBR1*03:01	99	61	0.588	0.620	0.536	**0.629**
DBR1*04:01	142	91	0.787	0.762	0.748	**0.863**
DBR1*07:01	94	72	0.800	0.728	0.732	**0.814**
DBR1*09:01	62	45	0.793	0.796	0.654	**0.889**
DBR1*11:01	94	72	0.614	0.629	**0.699**	0.657
DBR1*12:02	59	48	0.661	0.742	**0.824**	0.788
DBR1*13:01	57	47	0.538	0.472	0.591	**0.615**
DBR1*15:01	96	81	0.767	0.683	0.751	**0.799**
DBR1*15:02	54	41	0.760	0.735	0.730	**0.764**
Average			0.719	0.700	0.700	**0.770**

#### 3.4.4 Comparison with MHCAttnNet

We trained DeepMHCII with the cross-entropy objective function under the same 5-fold CV split over BD2020, where this objective function was used in MHCAttnNet (Venkatesh *et al.*, 2020), one of the competing methods of DeepMHCII. Table 5 shows the performance of DeepMHCII and MHCAttnNet, where following the original paper of MHCAttnNet, we used AUC over the whole test set, instead of the average AUC per MHC-II molecule. The AUC and accuracy of DeepMHCII were 4.1% and 3.0%, respectively, higher than those of MHCAttnNet, further demonstrating the performance advantage of DeepMHCII.

**Table 5.** Performance of DeepMHCII and MHCAttnNet on BD2020

Method	Accuracy	AUC
MHCAttnNet	0.755	0.758
DeepMHCII	**0.786**	**0.781**

### 3.5 Result analysis

In order to analyze (or to interpret) the experimental results of DeepMHCII, we conducted the following five experiments: (i) We compared DeepMHCII with DeepSeqPanII and NetMHCIIpan-3.2 in binding core prediction over BC2015. (ii) We visualized the binding motifs of MHC-II molecules by using sequence logos (Schneider and Stephens, 1990) and compared the sequence logos generated by DeepMHCII with those of DeepSeqPanII and NetMHCIIpan-3.2. (iii) To illustrate the biological perspective captured by DeepMHCII, we analyzed the weights (of nine pockets in the binding core) of BICL in DeepMHCII. (iv) We examined the performance of DeepMHCII under different kernel sizes (5–15). (v) We checked the time and space complexities of DeepMHCII, comparing with those of two competing methods, PUFFIN and DeepSeqPanII.

#### 3.5.1 Binding core prediction

The last column of Table 3 shows the results of predicting the binding core over BC2015 (more detailed results are shown in Supplementary Table S3). Note that PUFFIN cannot predict binding cores and thus is not shown in this column. Also note that NetMHCIIpan-3.2 has one variant, ‘NetMHCIIpan-3.2 (without offset)’, which uses the original prediction results to identify the binding core. Out of all 51 pairs, DeepMHCII correctly predicted 47, being followed by NetMHCIIpan-3.2 (45), NetMHCIIpan-3.2 without offset (28) and DeepSeqPanII (10). This result highlights the advantage of DeepMHCII of flexibly modeling the binding core and its high interpretability over competing methods.

#### 3.5.2 Sequence logos

**Fig. 4.**
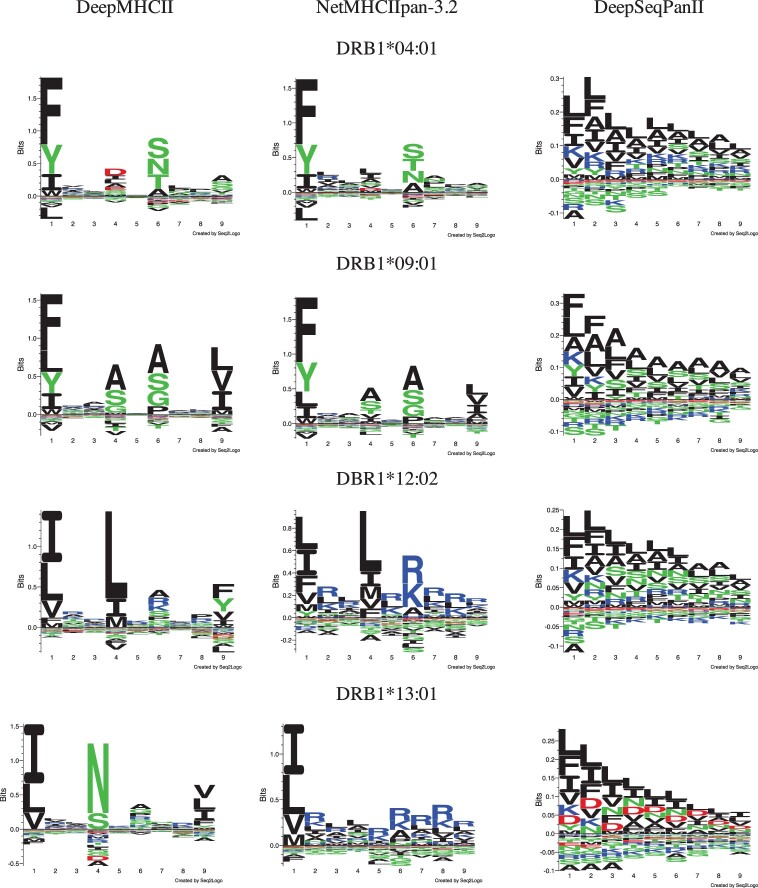
Sequence logos by DeepMHCII, NetMHCIIpan-3.2 and DeepSeqPanII

We visualized the binding motifs of MHC-II molecules obtained by each prediction method as sequence logos (Schneider and Stephens, 1990). Following the description in Nielsen *et al.* (2007), we first computed the binding scores of 100 000 random peptides from SwissProt and then selected the top 1% predicted binders to draw sequence logos (with default settings). Since PUFFIN does not have the ability to predict the binding core, we compared the sequence logos generated by DeepMHCII, NetMHCIIpan-3.2 and DeepSeqPanII. We focused on four MHC-II molecules, DRB1*04:01, DRB1*09:01, DRB1*12:02 and DRB1*13:01, in ID2017, where DeepMHCII outperformed NetMHCIIpan-3.2 most. Figure 4 shows the sequence logos of these four MHC-II molecules by different three methods. Each sequence logo has 1st to 9th positions (pockets) in the x-axis, where at each position, the total height [of letters (amino acids)] represents the relative information content (also importance) of the corresponding position in the motif, and the height of each letter shows the frequency of the corresponding amino acid in the position. It is widely observed and generally thought that P1 (pocket 1), P4, P6 and P9 are four primary anchors, which are most important for peptide binding (Rammensee *et al.*, 1999). All four sequence logos of DeepMHCII are consistent with this widely accepted understanding. In contrast, DeepSeqPanII could not distinguish these four primary anchors from other five pockets clearly, and the sequence logos by NetMHCIIpan3.2 contained more noise at pockets, especially non-primary pockets in DRB1*12:02 and DRB1*13:01, making frequent amino acids at each pocket totally unclear. Furthermore, by taking a closer look at primary anchors, we could observe clear differences among prediction methods in amino acid preference in primary anchors. For example, P4 of DRB1*04:01 was identified as a primary anchor by both DeepMHCII and NetMHCIIpan-3.2, where preferred amino acids in P4 by DeepMHCII were [DILAES] but [LIASVDM] by NetMHCIIpan-3.2. According to SYFPEITHI (Rammensee *et al.*, 1999), an MHC binding motif database, P4 allows amino acid E. This information is consistent with the sequence logo of DeepMHCII. Overall, DeepMHCII could generate better sequence logos than the competing methods.

#### 3.5.3 Analyzing weights of BICL

We examined the importance of each position in the binding core by checking the absolute value of the weight (obtained by BICL) for each position of the binding core. For each pocket, we summed the absolute weight values for each model and computed the mean and standard deviation from all *T *=* *20 models of DeepMHCII. Table 6 shows the mean and standard deviation of each pocket. The values of positions 1, 4, 6 and 9 (which are in boldface) were much larger than other positions, being consistent with that P1 (pocket 1), P4, P6 and P9 are primary anchors (Rammensee *et al.*, 1999). From this result, we can see that DeepMHCII could learn biological knowledge from data well, showing the validity of DeepMHCII from a biological perspective.

**Table 6.** Weights of BICL for nine pockets of the binding core

Pocket	Weight
P1	**243.21 **±** **10.14
P2	230.51 ± 10.22
P3	232.19 ± 8.48
P4	**256.36 **±** **10.48
P5	232.00 ± 10.86
P6	**251.38 **±** **10.02
P7	239.53 ± 9.44
P8	227.72 ± 9.80
P9	**253.76 **±** **10.55

#### 3.5.4 Ablation experiments

We examined DeepMHCII with the single kernel size *k*, selecting *k* from 5, 7, 9, 11, 13 and 15, where we call DeepMHCII trained by the kernel size of *k* as DeepMHCII_*k*_ (Note that original DeepMHCII uses four different kernel sizes: 9, 11, 13 and 15 at once). Table 7 reports the performance of 5-fold CV over BD2016 by DeepMHCII_*k*_. We have three findings: (i) DeepMHCII_9_ outperformed DeepMHCII_5_ and DeepMHCII_7_ significantly. Specifically, DeepMHCII_9_ achieved PCC of 0.676, which was followed by DeepMHCII_7_ (0.651) and DeepMHCII_5_ (0.637). This is consistent with that the standard binding core is with nine amino acids. (ii) DeepMHCII_11_, DeepMHCII_13_ and DeepMHCII_15_ achieved a similar performance, which was higher than DeepMHCII_9_. For example, both DeepMHCII_13_ and DeepMHCII_15_ achieved PCC of 0.686, which was higher than DeepMHCII_9_ (0.676). This suggests that peptide franking region has some positive effect on MHC-II peptide binding. (iii) DeepMHCII achieved the best performance with AUC of 0.856 and PCC of 0.691 among all compared methods. All these results confirm that the high performance of DeepMHCII was obtained by incorporating biological knowledge into the model design.

**Table 7.** Performance of DeepMHCII with different kernel sizes under 5-fold CV

Method	AUC	PCC
DeepMHCII _5_	0.828	0.637
DeepMHCII _7_	0.834	0.651
DeepMHCII _9_	0.849	0.676
DeepMHCII_11_	0.853	0.685
DeepMHCII_13_	0.854	0.686
DeepMHCII_15_	0.853	0.686
DeepMHCII	**0.856**	**0.691**

In addition, we compared DeepMHCII with a vanilla CNN, which uses a convolutional layer instead of BICL on the peptide and concatenates representations of the peptide and allele directly. As shown in Table 8, we can see that the performance of DeepMHCII is much better than CNN.

**Table 8.** Performance of DeepMHCII with CNN instead of BICL

Methods	AUC	PCC
CNN	0.806	0.608
DeepMHCII	**0.856**	**0.691**

#### 3.5.5 Time and space complexity

Table 9 shows the amount of time necessary for training and prediction of DeepMHCII and competing methods. All methods were run on a single Nvidia Titan X (pascal). The amount of time for training DeepMHCII is equivalent to that for training PUFFIN and is much smaller than that for training DeepSeqPanII, which is based on recurrent neural networks. More notably, for prediction, DeepMHCII is significantly faster than both PUFFIN and DeepSeqPanII. We further examined the size of The trained model between DeepMHCII and competing methods. As shown in the last column of the table, the size of DeepMHCII is only 1.4 Mbytes, which is far smaller than those of PUFFIN (24.1 Mbytes) and DeepSeqPanII (15.3 Mbytes). This is a practically sizable difference.

**Table 9.** Computation time and model size of DeepMHCII and competing methods

Method	Training (s)	Test (ms/sample)	Model size (MB)
PUFFIN	651	2.252	24.1
DeepSeqPanII	7967	0.828	15.3
DeepMHCII	693	0.583	1.4

## 4 Conclusion and discussion

We have proposed a new deep learning model, DeepMHCII, for predicting peptide-MHC binding affinity, the binding core and important pockets in the binding core. Considering the biological properties behind peptide-MHC binding, DeepMHCII has explicitly incorporated the interaction processes between a peptide and an MHC-II molecule through interaction convolution layers, which makes us biologically understand the peptide-MHC binding core and predict the binding affinity.

Extensive experiments with four large-scale datasets have demonstrated that DeepMHCII significantly outperformed all four state-of-the-art competing methods under a wide variety of settings. Furthermore, DeepMHCII captured the motifs more precisely than the compared methods, verifying the high performance in predicting the binding core and also the important pockets. All these results proved the usefulness of DeepMHCII in terms of the high accuracy but also precise biological discovery and high scientific interpretability. A limitation of our study might be that we focus on peptide binding prediction other than epitope prediction, where the MHC binding peptide must be recognized by TCR. Possible future work would be to incorporate more biological knowledge into our model design to develop high-performance deep learning methods for epitope prediction (Blass and Ott, 2021).

## Funding

This work was supported by National Natural Science Foundation of China [No. 61872094], Shanghai Municipal Science and Technology Major Project [No.2018SHZDZX01], ZJ Lab and Shanghai Center for Brain Science and Brain-Inspired Technology to S.Z.; the 111 Project [No. B18015], Shanghai Municipal Science and Technology Major Project [No. 2017SHZDZX01] and Information Technology Facility, CAS-MPG Partner Institute for Computational Biology, Shanghai Institute for Biological Sciences, Chinese Academy of Sciences to R.Y. and W.Q.; H.M. has been supported in part by MEXT KAKENHI [grant numbers: 19H04169, 20F20809, 21H05027 and 22H03645] and the AIPSE program of the Academy of Finland.

*Conflict of Interest*: none declared.

## Supplementary Material

btac225_Supplementary_Data

## References

[btac225-B1] Andreatta M. et al (2015) Accurate pan-specific prediction of peptide-MHC class II binding affinity with improved binding core identification. Immunogenetics, 67, 641–650. 26416257 10.1007/s00251-015-0873-y PMC4637192

[btac225-B2] Andreatta M. et al (2018) An automated benchmarking platform for MHC class II binding prediction methods. Bioinformatics, 34, 1522–1528. 29281002 10.1093/bioinformatics/btx820 PMC5925780

[btac225-B3] Arnold P.Y. et al (2002) The majority of immunogenic epitopes generate CD4+ T cells that are dependent on MHC class II-bound peptide-flanking residues. J. Immunol., 169, 739–749. 12097376 10.4049/jimmunol.169.2.739

[btac225-B4] Blass E. , Ott P.A. (2021) Advances in the development of personalized neoantigen-based therapeutic cancer vaccines. Nat. Rev. Clin. Oncol., 18, 215–229. 33473220 10.1038/s41571-020-00460-2 PMC7816749

[btac225-B5] Burley S.K. et al (2021) RCSB protein data bank: powerful new tools for exploring 3D structures of biological macromolecules for basic and applied research and education in fundamental biology, biomedicine, biotechnology, bioengineering and energy sciences. Nucleic Acids Res., 49, D437– D451. 33211854 10.1093/nar/gkaa1038 PMC7779003

[btac225-B6] Cheng J. et al (2021) BERTMHC: improved MHC–peptide class II interaction prediction with transformer and multiple instance learning. Bioinformatics, 37, 4172–4179. 34096999 10.1093/bioinformatics/btab422 PMC9502151

[btac225-B7] Guo L. et al (2013) MHC2SKpan: a novel kernel based approach for pan-specific MHC class II peptide binding prediction. BMC Genomics, 14, S11. 10.1186/1471-2164-14-S5-S11 PMC3852073 24564280

[btac225-B8] Holland C.J. et al (2013) Re-directing CD4+ T cell responses with the flanking residues of MHC class II-bound peptides: the core is not enough. Front. Immunol., 4, 172. 23847615 10.3389/fimmu.2013.00172 PMC3696884

[btac225-B9] Hu X. et al (2010) MetaMHC: a meta approach to predict peptides binding to MHC molecules. Nucleic Acids Res., 38, W474–W479. 20483919 10.1093/nar/gkq407 PMC2896142

[btac225-B10] Ioffe S. , Szegedy C. (2015) Batch normalization: accelerating deep network training by reducing internal covariate shift. In: International Conference on Machine Learning, Lille France July 6-11, 2015. PMLR, pp. 448–456.

[btac225-B11] Janeway C.A. et al (2005) Immunobiology: The Immune System in Health and Disease, 6th edn. Garland Science Publishing, New York.

[btac225-B12] Jensen K.K. et al (2018) Improved methods for predicting peptide binding affinity to MHC class II molecules. Immunology, 154, 394–406. 29315598 10.1111/imm.12889 PMC6002223

[btac225-B13] Karosiene E. et al (2013) NetMHCIIpan-3.0, a common pan-specific MHC class II prediction method including all three human MHC class II isotypes, HLA-DR, HLA-DP and HLA-DQ. Immunogenetics, 65, 711–724. 23900783 10.1007/s00251-013-0720-y PMC3809066

[btac225-B14] Liu Z. et al (2021) DeepSeqPanII: an interpretable recurrent neural network model with attention mechanism for peptide-HLA class II binding prediction. IEEE/ACM Trans. Comput. Biol. Bioinformatics, 1. https://doi.org/10.1109/TCBB.2021.3074927. 10.1109/TCBB.2021.3074927 33886473

[btac225-B15] Lund O. et al (2005). Immunological Bioinformatics. The MIT Press, Cambridge, MA, USA.

[btac225-B16] Mamitsuka H. (1998) Predicting peptides that bind to MHC molecules using supervised learning of hidden Markov models. Proteins Bioinformatics, 33, 460–474. 10.1002/(sici)1097-0134(19981201)33:4<460::aid-prot2>3.0.co;2-m 9849933

[btac225-B17] Nielsen M. et al (2007) Prediction of MHC class II binding affinity using SMM-align, a novel stabilization matrix alignment method. BMC Bioinformatics, 8, 238. 17608956 10.1186/1471-2105-8-238 PMC1939856

[btac225-B18] Nielsen M. et al (2010) MHC class II epitope predictive algorithms. Immunology, 130, 319–328. 20408898 10.1111/j.1365-2567.2010.03268.x PMC2913211

[btac225-B19] Paszke A. et al (2019) PyTorch: an imperative style, high-performance deep learning library. In: Wallach,H. *et al. *(eds.) Advances in Neural Information Processing systems, Vol. 32. Curran Associates, Inc.

[btac225-B20] Rammensee H.-G. et al (1999) SYFPEITHI: database for MHC ligands and peptide motifs. Immunogenetics, 50, 213–219. 10602881 10.1007/s002510050595

[btac225-B21] Schneider T.D. , Stephens R.M. (1990) Sequence logos: a new way to display consensus sequences. Nucleic Acids Res., 18, 6097–6100. 2172928 10.1093/nar/18.20.6097 PMC332411

[btac225-B22] Srivastava N. et al (2014) Dropout: a simple way to prevent neural networks from overfitting. J. Mach. Learn. Res., 15, 1929–1958.

[btac225-B23] Traherne J. (2008) Human MHC architecture and evolution: implications for disease association studies. Int. J. Immunogenet., 35, 179–192. 18397301 10.1111/j.1744-313X.2008.00765.x PMC2408657

[btac225-B24] Udaka K. et al (2002) Empirical evaluation of a dynamic experiment design method for prediction of MHC class I-binding peptides. J. Immunol., 69, 5744–5753. 10.4049/jimmunol.169.10.5744 12421954

[btac225-B25] Venkatesh G. et al (2020) MHCAttnNet: predicting MHC-peptide bindings for MHC alleles classes I and II using an attention-based deep neural model. Bioinformatics, 36, i399–i406. 32657386 10.1093/bioinformatics/btaa479 PMC7355292

[btac225-B26] Vita R. et al (2019) The immune epitope database (IEDB): 2018 update. Nucleic Acids Res., 47, D339–D343. 30357391 10.1093/nar/gky1006 PMC6324067

[btac225-B27] Xu Y. et al (2016) MetaMHCpan, a meta approach for pan-specific MHC peptide binding prediction. Methods Mol. Biol., 1404, 753–760. 27076335 10.1007/978-1-4939-3389-1_49

[btac225-B28] Zeiler M.D. (2012) Adadelta: an adaptive learning rate method. arXiv preprint arXiv:1212.5701. https://arxiv.org/abs/1212.5701.

[btac225-B29] Zeng H. , Gifford D.K. (2019) Quantification of uncertainty in peptide-MHC binding prediction improves high-affinity peptide selection for therapeutic design. Cell Syst., 9, 159–166. 31176619 10.1016/j.cels.2019.05.004 PMC6715517

[btac225-B30] Zhang L. et al (2012a) TEPITOPEpan: extending TEPITOPE for peptide binding prediction covering over 700 HLA-DR molecules. PLoS One, 7, e30483. 22383964 10.1371/journal.pone.0030483 PMC3285624

[btac225-B31] Zhang L. et al (2012b) Toward more accurate pan-specific MHC-peptide binding prediction: a review of current methods and tools. Brief. Bioinformatics, 13, 350–364. 21949215 10.1093/bib/bbr060

[btac225-B32] Zhu S. et al (2006) Improving MHC binding peptide prediction by incorporating binding data of auxiliary MHC molecules. Bioinformatics, 22, 1648–1655. 16613909 10.1093/bioinformatics/btl141

