# Supplementary material for: DeepMHCII: a novel binding core-aware deep interaction model for accurate MHC-II peptide binding affinity prediction
Source: Bioinformatics. 2022 Jun 27;38(Suppl 1):i220–8. doi: 10.1093/bioinformatics/btac225 (PMC9235502; doi:10.1093/bioinformatics/btac225)
Supplement: btac225_Supplementary_Data [file btac225_supplementary_data.pdf]

Supplementary Materials of DeepMHCII

Ronghui You[1], Wei Qu[1], Hiroshi Mamitsuka[2], and Shanfeng Zhu *[1]

[1]Institute of Science and Technology for Brain-Inspired Intelligence, Fudan University
[2]Bioinformatics Center, Institute for Chemical Research, Kyoto University

1 Experimental Results

1.1 Detailed Results of Five Fold Cross-Validation over BD2016

Table S1 shows the detailed results of DeepMHCII and competing methods under 5-CV for all MHC-II molecules. DeepMHCII achieved the best AUC and PCC on 45 and 48 out of 61 MHC-II molecules, respectively. In addition, DeepMHCII achieved the highest average AUC (0.856) and PCC (0.691).

1.2 Detailed Results of LOMO over BD2016

Table S2 shows the detailed results of DeepMHCII and competing methods under LOMO for all MHC-II molecules. Similar to the results of five fold cross-validation, DeepMHCII achieved the the highest average AUC (0.817) and PCC (0.621). Specifically, DeepMHCII achieved the best AUC and PCC on 45 and 48 out of 61 MHC-II molecules, respectively.

1.3 Detailed Results of Strict LOMO over BD2016

Table S3 shows the detailed results of DeepMHCII and competing methods (used a bagging ensemble of 4 models) under strict LOMO for all MHC-II molecules. In this strict LOMO, for each test MHC molecule, we removed the most similar molecules from its training sets to avoid "easy" predictions. The distance to estimated the similarity is defined as $d(A,B) = \frac{s(A,B)}{\sqrt{s(A,A)\cdot s(B,B)}}$, where $s(A,B)$ is the BLOSUM50 alignment score between the pseudo sequences for MHC molecules A and B, respectively [1, 2]. A similarity threshold of 0.15 was used: All MHC molecules with a distance < 0.15 were removed for a given molecule. DeepMHCII achieved higher average AUC (0.747) and PCC (0.493). Specifically, DeepMHCII achieved better AUC and PCC on 39 and 40 out of 61 MHC-II molecules, respectively.

1.4 Binding Core Prediction over BC2015

Table S4 shows the detailed results of DeepMHCII, DeepSeqPanII and NetMHCIIpan 3.2 over BC2015, where PUFFIN could not predict the binding core and it's not be shown. For each pair (row) of an allele (MHC-II molecule) and antigen (peptide), red letters show the true binding core. Only the wrongly predicted binding core is shown, and the correctly predicted entry is blank, meaning that a method with a larger number of blank entries is better. Out of all 51 pairs, DeepMHCII correctly predicted 47, being followed by NetMHCIIpan-3.2 (45) and DeepSeqPanII (10).

*Corresponding author: zhusf@fudan.edu.cn

Table S1: Detailed five-fold cross-validation performance of DeepMHCII and competing methods.

Allele	NetMHCIIpan-3.2		PUFFIN		DeepSeqPanII		DeepMHCII	
	AUC	PCC	AUC	PCC	AUC	PCC	AUC	PCC
DRB1_0101	0.832	0.674	0.827	0.661	0.791	0.568	**0.840**	**0.687**
DRB1_0103	**0.678**	**0.408**	0.658	0.364	0.566	0.387	0.638	0.217
DRB1_0301	0.816	0.621	0.815	0.612	0.670	0.356	**0.832**	**0.638**
DRB1_0401	0.809	0.613	0.805	0.600	0.730	0.467	**0.818**	**0.629**
DRB1_0402	**0.701**	**0.526**	0.666	0.410	0.659	0.470	0.673	0.511
DRB1_0403	0.841	0.615	0.805	0.568	0.737	0.461	**0.897**	**0.706**
DRB1_0404	0.812	0.652	0.811	0.643	0.768	0.568	**0.818**	**0.663**
DRB1_0405	0.827	0.650	0.832	0.660	0.757	0.518	**0.847**	**0.685**
DRB1_0701	0.875	0.733	0.869	0.723	0.827	0.634	**0.887**	**0.759**
DRB1_0801	0.844	0.671	0.856	0.687	0.800	0.588	**0.860**	**0.693**
DRB1_0802	0.834	0.663	0.833	0.658	0.803	0.594	**0.843**	**0.676**
DRB1_0901	0.833	0.666	0.832	0.658	0.787	0.571	**0.841**	**0.678**
DRB1_1001	0.923	0.761	0.918	0.770	0.900	0.680	**0.923**	**0.781**
DRB1_1101	0.864	0.728	0.859	0.727	0.799	0.594	**0.873**	**0.746**
DRB1_1201	0.868	0.727	0.880	0.752	0.805	0.622	**0.890**	**0.768**
DRB1_1301	**0.857**	**0.658**	0.843	0.649	0.794	0.528	0.821	0.612
DRB1_1302	0.885	0.761	0.884	0.757	0.757	0.555	**0.885**	**0.764**
DRB1_1501	0.834	0.666	0.838	0.672	0.789	0.571	**0.847**	**0.693**
DRB1_1602	0.883	0.767	0.894	**0.788**	0.857	0.672	**0.895**	0.787
DRB3_0101	0.888	0.787	**0.904**	0.803	0.751	0.521	0.903	**0.804**
DRB3_0202	0.869	0.793	0.880	0.800	0.742	0.558	**0.888**	**0.812**
DRB3_0301	0.840	0.665	0.833	0.649	0.765	0.506	**0.845**	**0.705**
DRB4_0101	0.822	0.638	0.826	0.651	0.731	0.493	**0.841**	**0.682**
DRB4_0103	**0.841**	**0.629**	0.822	0.588	0.806	0.562	0.839	0.612
DRB5_0101	0.849	0.709	0.852	0.717	0.764	0.548	**0.864**	**0.735**
H-2-IAb	0.894	0.720	0.882	0.706	0.753	0.470	**0.898**	**0.728**
H-2-IAd	0.819	0.658	0.796	0.623	0.752	0.539	**0.839**	**0.678**
H-2-IAk	**0.635**	0.222	0.498	**0.240**	0.435	-0.030	0.541	0.218
H-2-IAs	**0.825**	**0.587**	0.772	0.496	0.479	0.112	0.814	0.580
H-2-IAu	0.765	0.545	0.822	**0.574**	0.786	0.430	**0.822**	0.570
H-2-IEd	0.754	**0.560**	**0.774**	0.528	0.686	0.424	0.740	0.510
H-2-IEk	**0.853**	**0.768**	0.846	0.759	0.741	0.582	0.821	0.736
HLA-DPA10103-DPB10201	0.917	0.741	**0.921**	0.736	0.832	0.583	0.919	**0.755**
HLA-DPA10103-DPB10301	0.902	0.783	0.894	0.771	0.842	0.677	**0.910**	**0.792**
HLA-DPA10103-DPB10401	0.935	0.884	0.933	0.881	0.879	0.775	**0.942**	**0.892**
HLA-DPA10103-DPB10402	0.710	0.425	0.738	0.395	0.639	0.415	**0.809**	**0.519**
HLA-DPA10103-DPB10601	0.995	**0.962**	0.993	0.955	0.972	0.912	**0.995**	0.957
HLA-DPA10201-DPB10101	0.903	0.844	0.900	0.843	0.854	0.754	**0.912**	**0.854**
HLA-DPA10201-DPB10501	0.911	0.804	0.911	0.804	0.874	0.741	**0.914**	**0.810**
HLA-DPA10201-DPB11401	0.930	0.836	0.937	0.851	0.881	0.744	**0.946**	**0.860**
HLA-DPA10301-DPB10402	0.904	0.837	0.903	0.836	0.856	0.754	**0.910**	**0.844**
HLA-DQA10101-DQB10501	0.900	0.781	0.909	0.798	0.724	0.467	**0.911**	**0.799**
HLA-DQA10102-DQB10501	0.839	0.654	**0.849**	**0.660**	0.751	0.491	0.824	0.627
HLA-DQA10102-DQB10502	0.835	0.654	0.857	0.665	0.720	0.460	**0.859**	**0.685**
HLA-DQA10102-DQB10602	0.890	0.798	0.877	0.779	0.827	0.679	**0.906**	**0.823**
HLA-DQA10103-DQB10603	0.861	0.577	0.851	0.578	0.803	0.463	**0.883**	**0.614**
HLA-DQA10104-DQB10503	0.805	0.596	0.830	0.620	0.686	0.405	**0.843**	**0.666**
HLA-DQA10201-DQB10202	0.814	0.614	0.837	0.625	0.642	0.408	**0.850**	**0.663**
HLA-DQA10201-DQB10301	0.849	0.649	0.852	0.660	0.795	0.561	**0.873**	**0.695**
HLA-DQA10201-DQB10303	0.894	0.736	0.891	0.724	0.815	0.593	**0.900**	**0.755**
HLA-DQA10201-DQB10402	0.860	0.634	0.861	0.642	0.714	0.373	**0.875**	**0.656**
HLA-DQA10301-DQB10301	**0.839**	**0.664**	0.814	0.623	0.735	0.501	0.815	0.636
HLA-DQA10301-DQB10302	0.810	0.657	0.847	0.697	0.650	0.388	**0.847**	**0.700**
HLA-DQA10303-DQB10402	0.820	0.543	0.837	**0.601**	0.671	0.262	**0.859**	0.592
HLA-DQA10401-DQB10402	0.883	0.759	**0.893**	**0.781**	0.813	0.609	0.879	0.746
HLA-DQA10501-DQB10201	0.876	0.759	0.881	0.770	0.780	0.585	**0.883**	**0.778**
HLA-DQA10501-DQB10301	0.915	0.786	0.913	0.783	0.758	0.488	**0.928**	**0.815**
HLA-DQA10501-DQB10302	0.822	0.638	**0.849**	**0.665**	0.792	0.542	0.844	0.650
HLA-DQA10501-DQB10303	0.876	0.682	0.875	0.706	0.820	0.566	**0.887**	**0.712**
HLA-DQA10501-DQB10402	0.868	**0.692**	**0.871**	0.691	0.713	0.380	0.870	0.684
HLA-DQA10601-DQB10402	**0.848**	0.568	0.831	**0.579**	0.671	0.264	0.836	0.530
Average	0.847	0.679	0.846	0.676	0.759	0.524	**0.856**	**0.691**
p-value	7.67e-6	2.72e-5	9.62e-8	7.67e-6	8.67e-19	5.38e-17		

Table S2: Detailed LOMO performance of DeepMHCII and competing methods.

Allele	NetMHCIIpan-3.2		PUFFIN		DeepSeqPanII		DeepMHCII	
	AUC	PCC	AUC	PCC	AUC	PCC	AUC	PCC
DRB1_0101	0.783	0.558	0.760	0.511	0.772	0.527	**0.800**	**0.600**
DRB1_0103	0.711	**0.458**	**0.757**	0.379	0.553	0.422	0.704	0.260
DRB1_0301	0.699	0.400	0.632	0.272	0.630	0.279	**0.745**	**0.481**
DRB1_0401	0.766	0.517	0.760	0.509	0.714	0.435	**0.782**	**0.558**
DRB1_0402	**0.789**	**0.646**	0.667	0.415	0.656	0.413	0.664	0.498
DRB1_0403	0.862	0.679	0.835	0.660	0.749	0.520	**0.889**	**0.725**
DRB1_0404	0.791	0.598	0.787	0.583	0.762	0.554	**0.808**	**0.631**
DRB1_0405	0.799	0.596	0.784	0.576	0.751	0.506	**0.813**	**0.620**
DRB1_0701	0.830	0.634	0.823	0.628	0.816	0.611	**0.840**	**0.666**
DRB1_0801	0.804	0.600	0.781	0.541	0.791	0.571	**0.816**	**0.603**
DRB1_0802	0.765	0.510	0.756	0.500	**0.794**	**0.570**	0.787	0.556
DRB1_0901	0.791	0.586	0.780	0.561	0.774	0.543	**0.804**	**0.612**
DRB1_1001	0.905	0.698	0.890	0.699	0.894	0.664	**0.913**	**0.743**
DRB1_1101	0.767	0.507	0.772	0.527	**0.782**	**0.561**	0.771	0.531
DRB1_1201	0.800	0.592	0.796	0.609	0.757	0.571	**0.824**	**0.653**
DRB1_1301	0.731	0.413	0.735	0.431	**0.780**	**0.494**	0.720	0.400
DRB1_1302	0.701	0.437	0.661	0.340	**0.728**	**0.510**	0.664	0.354
DRB1_1501	0.780	0.551	**0.796**	0.579	0.784	0.558	0.794	**0.585**
DRB1_1602	0.866	0.731	0.862	0.711	0.858	0.665	**0.880**	**0.752**
DRB3_0101	0.801	0.583	0.809	0.639	0.641	0.299	**0.832**	**0.666**
DRB3_0202	0.756	0.596	0.770	0.600	0.738	0.528	**0.793**	**0.649**
DRB3_0301	0.734	0.431	0.766	0.516	0.756	0.479	**0.792**	**0.589**
DRB4_0101	**0.726**	**0.462**	0.681	0.390	0.703	0.451	0.715	0.452
DRB4_0103	0.794	0.513	0.764	0.460	**0.802**	**0.548**	0.780	0.483
DRB5_0101	0.765	0.553	0.763	0.538	0.751	0.525	**0.782**	**0.578**
H-2-IAb	**0.780**	**0.557**	0.770	0.544	0.681	0.348	0.756	0.487
H-2-IAd	0.725	0.461	0.663	0.334	0.724	0.490	**0.761**	**0.523**
H-2-IAk	0.383	-0.102	0.412	-0.080	**0.426**	**-0.074**	0.401	-0.092
H-2-IAs	**0.514**	**0.195**	0.435	0.066	0.476	0.085	0.425	0.007
H-2-IAu	0.739	0.475	**0.861**	**0.544**	0.774	0.410	0.793	0.534
H-2-IEd	0.646	0.356	0.695	0.401	**0.699**	**0.407**	0.664	0.385
H-2-IEk	0.854	0.716	**0.859**	**0.716**	0.733	0.555	0.792	0.660
HLA-DPA10103-DPB10201	0.882	0.669	0.899	0.696	0.810	0.536	**0.905**	**0.716**
HLA-DPA10103-DPB10301	0.840	0.672	0.852	0.700	0.790	0.600	**0.857**	**0.697**
HLA-DPA10103-DPB10401	0.921	0.863	0.918	0.858	0.851	0.723	**0.928**	**0.872**
HLA-DPA10103-DPB10402	0.719	0.462	0.744	0.432	0.627	0.396	**0.809**	**0.519**
HLA-DPA10103-DPB10601	**0.993**	0.945	0.990	0.943	0.971	0.909	0.990	**0.950**
HLA-DPA10201-DPB10101	**0.880**	**0.815**	0.875	0.815	0.833	0.710	0.874	0.807
HLA-DPA10201-DPB10501	**0.880**	**0.756**	0.866	0.736	0.864	0.715	0.872	0.750
HLA-DPA10201-DPB11401	0.896	0.751	0.895	0.764	0.821	0.645	**0.915**	**0.775**
HLA-DPA10301-DPB10402	0.889	0.805	0.886	0.807	0.842	0.727	**0.893**	**0.816**
HLA-DQA10101-DQB10501	0.678	0.396	0.669	0.360	0.606	0.239	**0.755**	**0.525**
HLA-DQA10102-DQB10501	0.618	0.224	0.589	0.181	**0.740**	**0.474**	0.578	0.175
HLA-DQA10102-DQB10502	0.694	0.398	0.688	0.399	0.652	0.349	**0.723**	**0.466**
HLA-DQA10102-DQB10602	0.786	0.561	**0.802**	**0.616**	0.776	0.569	0.777	0.573
HLA-DQA10103-DQB10603	0.837	0.508	0.836	0.526	0.786	0.443	**0.867**	**0.559**
HLA-DQA10104-DQB10503	0.765	0.534	0.779	0.555	0.628	0.321	**0.823**	**0.631**
HLA-DQA10201-DQB10202	0.789	0.567	0.747	0.506	0.589	0.336	**0.800**	**0.605**
HLA-DQA10201-DQB10301	0.835	0.620	0.836	0.625	0.749	0.468	**0.842**	**0.641**
HLA-DQA10201-DQB10303	0.873	0.714	0.880	0.704	0.808	0.569	**0.885**	**0.733**
HLA-DQA10201-DQB10402	0.840	0.590	0.825	0.563	0.707	0.357	**0.861**	**0.628**
HLA-DQA10301-DQB10301	**0.814**	**0.628**	0.755	0.508	0.725	0.482	0.802	0.594
HLA-DQA10301-DQB10302	0.648	0.379	**0.713**	**0.481**	0.603	0.308	0.661	0.405
HLA-DQA10303-DQB10402	0.680	0.268	0.645	0.198	0.675	0.273	**0.747**	**0.393**
HLA-DQA10401-DQB10402	0.666	0.297	0.586	0.169	**0.789**	**0.552**	0.659	0.290
HLA-DQA10501-DQB10201	**0.779**	**0.591**	0.764	0.572	0.662	0.363	0.762	0.574
HLA-DQA10501-DQB10301	0.800	0.575	0.799	0.578	0.582	0.160	**0.823**	**0.615**
HLA-DQA10501-DQB10302	0.802	0.610	0.834	0.626	0.779	0.506	**0.817**	**0.631**
HLA-DQA10501-DQB10303	0.864	0.677	0.862	0.673	0.797	0.532	**0.870**	**0.684**
HLA-DQA10501-DQB10402	**0.824**	**0.609**	0.823	0.596	0.692	0.341	0.767	0.507
HLA-DQA10601-DQB10402	0.636	0.181	0.607	0.119	0.662	0.248	**0.711**	**0.302**
Average	0.775	0.544	0.768	0.525	0.732	0.473	**0.785**	**0.560**
p-value	1.87e-3	2.64e-4	4.44e-3	2.72e-5	9.62e-8	4.59e-7		

Table S3: Detailed Strict LOMO performance of DeepMHCII and PUFFIN.

Allele	PUFFIN		DeepMHCII	
	AUC	PCC	AUC	PCC
DRB1_0101	0.776	0.554	**0.797**	**0.593**
DRB1_0103	0.559	**0.329**	**0.691**	0.242
DRB1_0301	0.654	0.316	**0.668**	**0.339**
DRB1_0401	0.741	0.482	**0.761**	**0.496**
DRB1_0402	**0.724**	**0.558**	0.676	0.439
DRB1_0403	0.746	0.356	**0.819**	**0.538**
DRB1_0404	0.730	0.506	**0.770**	**0.574**
DRB1_0405	0.756	0.523	**0.781**	**0.555**
DRB1_0701	0.825	0.629	**0.832**	**0.659**
DRB1_0801	**0.755**	0.485	0.749	**0.494**
DRB1_0802	**0.763**	**0.515**	0.762	0.497
DRB1_0901	0.764	0.528	**0.779**	**0.562**
DRB1_1001	0.888	0.693	**0.907**	**0.701**
DRB1_1101	0.783	0.569	**0.790**	**0.592**
DRB1_1201	0.745	0.541	**0.820**	**0.640**
DRB1_1301	0.768	0.510	**0.802**	**0.553**
DRB1_1302	**0.744**	0.488	0.710	**0.449**
DRB1_1501	0.780	0.548	**0.782**	**0.555**
DRB1_1602	0.840	0.702	**0.859**	**0.718**
DRB3_0101	**0.717**	**0.437**	0.707	0.424
DRB3_0202	0.730	0.504	**0.740**	**0.526**
DRB3_0301	0.715	0.436	**0.744**	**0.488**
DRB4_0101	0.629	0.305	**0.716**	**0.456**
DRB4_0103	**0.804**	**0.555**	0.798	0.510
DRB5_0101	0.763	0.548	**0.775**	**0.565**
H-2-IAb	**0.760**	**0.523**	0.740	0.457
H-2-IAd	0.628	0.276	**0.741**	**0.499**
H-2-IAk	0.383	**-0.141**	**0.394**	-0.149
H-2-IAs	**0.426**	**0.016**	0.397	-0.024
H-2-IAu	0.739	**0.440**	**0.765**	0.426
H-2-IEd	**0.704**	0.383	0.680	**0.403**
H-2-IEk	**0.829**	0.655	0.798	**0.657**
HLA-DPA10103-DPB10201	**0.846**	**0.615**	0.821	0.552
HLA-DPA10103-DPB10301	0.626	0.333	**0.675**	**0.411**
HLA-DPA10103-DPB10401	**0.854**	0.727	0.847	**0.760**
HLA-DPA10103-DPB10402	**0.679**	**0.399**	0.623	0.346
HLA-DPA10103-DPB10601	**0.994**	**0.957**	0.991	0.934
HLA-DPA10201-DPB10101	0.880	0.810	**0.887**	**0.813**
HLA-DPA10201-DPB10501	**0.855**	0.683	0.854	**0.708**
HLA-DPA10201-DPB11401	0.665	0.460	**0.781**	**0.593**
HLA-DPA10301-DPB10402	**0.867**	**0.775**	0.855	0.769
HLA-DQA10101-DQB10501	0.653	0.319	**0.753**	**0.486**
HLA-DQA10102-DQB10501	0.693	0.397	**0.693**	**0.397**
HLA-DQA10102-DQB10502	**0.677**	**0.378**	0.668	0.363
HLA-DQA10102-DQB10602	0.693	0.403	**0.761**	**0.535**
HLA-DQA10103-DQB10603	0.760	0.474	**0.813**	**0.497**
HLA-DQA10104-DQB10503	**0.628**	**0.348**	0.621	0.342
HLA-DQA10201-DQB10202	0.665	0.428	**0.680**	**0.462**
HLA-DQA10201-DQB10301	0.755	0.479	**0.768**	**0.501**
HLA-DQA10201-DQB10303	**0.863**	**0.652**	0.844	0.641
HLA-DQA10201-DQB10402	0.721	**0.379**	**0.734**	0.378
HLA-DQA10301-DQB10301	0.694	0.406	**0.710**	**0.492**
HLA-DQA10301-DQB10302	**0.685**	**0.415**	0.655	0.366
HLA-DQA10303-DQB10402	0.537	0.058	**0.608**	**0.118**
HLA-DQA10401-DQB10402	0.751	0.487	**0.758**	**0.504**
HLA-DQA10501-DQB10201	0.686	**0.433**	**0.690**	0.432
HLA-DQA10501-DQB10301	0.604	0.213	**0.637**	**0.283**
HLA-DQA10501-DQB10302	**0.829**	0.586	0.794	**0.597**
HLA-DQA10501-DQB10303	0.819	0.599	**0.835**	**0.612**
HLA-DQA10501-DQB10402	0.702	0.366	**0.748**	**0.441**
HLA-DQA10601-DQB10402	0.654	0.220	**0.706**	**0.291**
Average	0.730	0.468	**0.747**	**0.493**
p-value	0.0396	0.0204		

Table S4: Binding core prediction results. Red letters are true cores. Only wrongly predicted cores are shown for each method.

PDB	Allele	Antigen	DeepSeqPanII	NetMHCIIpan-3.2 (without offset)	NetMHCIIpan-3.2 (with offset)	DeepMHCII
2FSE	DRB1*01:01	AGFKGEQGPKGEPG	KGEQGPKGE			
1J8H	DRB1*04:01	PKYVKQNTLKLAT	VKQNTLKLA			
1FYT	DRB1*01:01	PKYVKQNTLKLAT	VKQNTLKLA			
3L6F	DRB1*01:01	APPAYEKLSAEQSPP	AYEKLSAEQ			
2Q6W	DRB3*01:01	AWRSDEALPLGS	SDEALPLGS			
1A6A	DRB1*03:01	PVSKMRMATPLLMQA	KMRMATPLL			
2IPK	DRB1*01:01	XPKWVKQNTLKLAT				
1SJH	DRB1*01:01	PEVIPMFSALSEG	IPMFSALSE			
4H1L	DRB3*03:01	QHIRCNIPKRISA	RCNIPKRIS			
3QXA	DRB1*01:01	PVSKMRMATPLLMQA	KMRMATPLL			
3PGD	DRB1*01:01	KMRMATPLLMQALPM	MATPLLMQA			
3PDO	DRB1*01:01	KPVSKMRMATPLLMQALPM	SKMRMATPL			
1AQD	DRB1*01:01	VGSDWRFLRGYHQYA	DWRFLRGYH			
1PYW	DRB1*01:01	XFVKQNAAALX	VKQNAAALX			
1KLG	DRB1*01:01	GELIGTLNAAKVPAD				
3C5J	DRB3*03:01	QVIILNHPGQISA	ILNHPGQIS			
4H26	DRB3*03:01	QWIRVNIPKRI				
4OV5	DRB1*01:01	GSDARFLRGYHLYA				
4IS6	DRB1*04:01	WNRQLYPEWTEAQRLD	QLYPEWTEA			
4H25	DRB3*03:01	QHIRCNIPKRIGPSKVATLVPR	RCNIPKRIG			
1SJE	DRB1*01:01	PEVIPMFSALSEGATP	IPMFSALSE			
1H15	DRB5*01:01	GGVYHFVKKHVHES				
1T5X	DRB1*01:01	AAYSDQATPLLLSPR	SDQATPLLL			
1BX2	DRB1*15:01	ENPVVHFFKNIVTPR	VVHFFKNIV	VVHFFKNIV		
4MD4	DRB1*04:01	ATEYRVRVNSAYQDK				
2SEB	DRB1*04:01	AYMRADAAAGGA	RADAAAGGA	YMRADAAAG	YMRADAAAG	
1ZGL	DRB5*01:01	VHFFKNIVTPRTPGG		FFKNIVTPR		
4I5B	DRB1*01:01	VVKQNCLKLATK	QNCLKLATK	VKQNCLKLA	VKQNCLKLA	QNCLKLATK
4MD5	DRB1*04:04	SAVRLRSSVPGVR	RLRSSVPGV			
1HQR	DRB5*01:01	VHFFKNIVTPRTP		FFKNIVTPR		
4MCZ	DRB1*04:01	GVYATRSSAVRLR	ATRSSAVRL	VYATRSSAV	VYATRSSAV	VYATRSSAV
4MCY	DRB1*04:01	SAVRLRSSVPGVR	RLRSSVPGV			
1FV1	DRB5*01:01	NPVVHFFKNIVTPRTPPPSQ	VHFFKNIVT	FFKNIVTPR		
1YMM	DRB1*15:01	ENPVVHFFKNIVTPRGGSGGGGG	VVHFFKNIV	VVHFFKNIV		
4AEN	DRB1*01:01	MPLAQMLLPTAMRMKM	AQMLLPTAM			
1JK8	DQA1*03:03-DQB1*03:02	LVEALYLVCGERGG	ALYLVCGER	MRMATPLLM		
1S9V	DQA1*05:05-DQB1*02:01	LQPFPQPELPY		SQAVHAAHA	LQPFPQPEL	QPFPQPELP
1UVQ	DQA1*01:02-DQB1*06:02	MNLPSTKVSWAAVGGGGSLV	PSTKVSWAA	PELPYPQPG	TKVSWAAVG	
4GG6	DQA1*03:01-DQB1*03:02	QQYPSGEGSFQPSQENPQ	PSGEGSFQP	AQKAKANKA		
4D8P	DQA1*03:01-DQB1*02:01	PQPEQPEQPFPQP		LQPFPQPEL		PQPEQPEQP
4OZG	DQA1*05:05-DQB1*02:01	APQPELPYPQPGS	PELPYPQPG	TQGVTAASS	PELPYPQPG	
4P4K	DPA1*01:03-DPB1*02:01	QAFWIDLFETIG	WIDLFETIG	KVTVAFNQF		
4P57	DPA1*01:03-DPB1*02:01	QAFWIDLFETIGGGSLV	WIDLFETIG	TKVSWAAVG		
3LQZ	DPA1*01:03-DPB1*02:01	RKFHYLPFLPSTGGS	HYLPFLPST	EGSFQPSQE		
3WEX	DPA1*02:01-DPB1*05:01	KVTVAFNQFGGS	VAFNQFGGS	EALYLVCGE		
4P5M	DPA1*01:03-DPB1*02:01	QAYDGKDYIALKG	DGKDYIALK	EQPEQPFPQ		
1MUJ	H-2-IAb	PVSKMRMATPLLMQA	KMRMATPLL	FWIDLFETI		
4P23	H-2-IAb	FEAQKAKANKAVD	QKAKANKAV	FWIDLFETI		
1IAO	H-2-IAd	ISQAVHAAHAEI	AVHAAHAEI	FHYLPFLPS		
2IAD	H-2-IAd	HATQGVTAASSHE	QGVTAASSH	YDGKDYIAL		
			10/51	28/51	45/51	47/51

References

[1] K. K. Jensen, M. Andreatta, P. Marcatili, S. Buus, J. A. Greenbaum, Z. Yan, A. Sette, B. Peters, and M. Nielsen. Improved methods for predicting peptide binding affinity to MHC class II molecules. *Immunology*, 154(3):394–406, 2018.

[2] S. Henikoff and J. G. Henikoff. Amino acid substitution matrices from protein blocks. *Proceedings of the National Academy of Sciences of the United States of America*, 89 22:10915–9, 1992.
